# Empirical versus estimated accuracy of imputation: optimising filtering thresholds for sequence imputation

**DOI:** 10.1186/s12711-024-00942-2

**Published:** 2024-11-15

**Authors:** Tuan V. Nguyen, Sunduimijid Bolormaa, Coralie M. Reich, Amanda J. Chamberlain, Christy J. Vander Jagt, Hans D. Daetwyler, Iona M. MacLeod

**Affiliations:** 1 Agriculture Victoria, Centre for AgriBiosciences, AgriBio, Bundoora, VIC 3083 Australia; 2 School of Applied Systems Biology, La Trobe University, Bundoora, VIC 3083 Australia

## Abstract

**Background:**

Genotype imputation is a cost-effective method for obtaining sequence genotypes for downstream analyses such as genome-wide association studies (GWAS). However, low imputation accuracy can increase the risk of false positives, so it is important to pre-filter data or at least assess the potential limitations due to imputation accuracy. In this study, we benchmarked three different imputation programs (Beagle 5.2, Minimac4 and IMPUTE5) and compared the empirical accuracy of imputation with the software estimated accuracy of imputation (Rsq_soft_). We also tested the accuracy of imputation in cattle for autosomal and X chromosomes, SNP and INDEL, when imputing from either low-density or high-density genotypes.

**Results:**

The accuracy of imputing sequence variants from real high-density genotypes was higher than from low-density genotypes. In our software benchmark, all programs performed well with only minor differences in accuracy. While there was a close relationship between empirical imputation accuracy and the imputation Rsq_soft_, this differed considerably for Minimac4 compared to Beagle 5.2 and IMPUTE5. We found that the Rsq_soft_ threshold for removing poorly imputed variants must be customised according to the software and this should be accounted for when merging data from multiple studies, such as in meta-GWAS studies. We also found that imposing an Rsq_soft_ filter has a positive impact on genomic regions with poor imputation accuracy due to large segmental duplications that are susceptible to error-prone alignment. Overall, our results showed that on average the imputation accuracy for INDEL was approximately 6% lower than SNP for all software programs. Importantly, the imputation accuracy for the non-PAR (non-Pseudo-Autosomal Region) of the X chromosome was comparable to autosomal imputation accuracy, while for the PAR it was substantially lower, particularly when starting from low-density genotypes.

**Conclusions:**

This study provides an empirically derived approach to apply customised software-specific Rsq_soft_ thresholds for downstream analyses of imputed variants, such as needed for a meta-GWAS. The very poor empirical imputation accuracy for variants on the PAR when starting from low density genotypes demonstrates that this region should be imputed starting from a higher density of real genotypes.

**Supplementary Information:**

The online version contains supplementary material available at 10.1186/s12711-024-00942-2.

## Background

Imputation is the process of predicting missing genome-wide genotypes in individuals with lower density genotypes by utilizing a reference population that has higher density genotypes [1, 2]. In recent years, the availability of imputed sequence data for downstream analyses such as genome-wide association studies (GWAS), has become extremely useful to explore the underlying genetic mechanisms of various phenotypes (e.g. [3–5]). Furthermore, candidate causal variants identified in imputed sequence GWAS can contribute to improve the accuracy of genomic prediction of complex traits in livestock (e.g. [6–8]). However, sequence GWAS often have low statistical power to detect many of the variants that affect complex traits because the majority of effects are small [9]. Thus, research has now shifted towards large-scale coordinated meta-GWAS to improve statistical power [10, 11], for example in humans [12, 13], plants [14, 15], and livestock [16–18]. A challenge of these meta-analyses is quality control to filter out poorly imputed variants, particularly when contributors have used different imputation software.

Two popular algorithmic approaches for genotype imputation are family- or population-based. In brief, family imputation utilizes relationships and shared genetic segments whilst population imputation uses large reference panels and linkage disequilibrium patterns. Some imputation programs combine both population- and family-based approaches and these are popular for use in livestock (for example AlphaImpute [19], FindHap [20], FImpute [21]). However, most of these programs do not offer an internally calculated imputation quality measure (Rsq_soft_) that is available in some other popular imputation software. At sequence level, an Rsq_soft_ estimate of the imputation accuracy is an indispensable quality control tool for removing poorly imputed variants from downstream analysis such as GWAS [1]. In general, several factors influence the final quality of imputation including: the choice of software, initial starting genotype density, reference population size and their relatedness to the target individuals [22]. Furthermore, the ever-increasing number of genotyped individuals and sequence reference individuals presents computational challenges for imputation, resulting in algorithmic modifications to improve computational efficiency of software. While several studies in cattle over the past decade have reported the accuracy of imputing from SNP panels to sequence variants [23–25], further studies on sequence imputation benchmarking are required for several reasons. First, it is critical to benchmark the internal software estimate of imputation accuracy versus empirically assessed imputation accuracy across different software. This provides an evaluation of how closely the software estimate mirrors the empirical accuracy and determines if this relationship differs between software. Second, to the best of our knowledge, no cattle study has reported the effectiveness of INDEL versus SNP imputation at sequence level using the updated reference genome ARS-UCD 1.2 [26], even though INDEL account for around 7% to 10% of all variants [27]. Third, this is the first study to compare sequence imputation accuracy for autosomal accuracy versus the pseudo-autosomal region (PAR) and non-PAR of the X chromosome from both low and high density SNP panels to sequence using the ARS-UCD 1.2 [26].

In this study, we evaluated the relationship between the software estimated accuracy of imputation (Rsq_soft_) and the empirical accuracy of imputation. We did this for both SNP and INDEL using three popular and competitive software packages: Beagle 5.2 [28], Minimac4 [29], and IMPUTE5 [30]. Results are compared for imputing either from Low Density (LD, ~ 7000 variants) or from High Density SNP array genotypes (HD, ~ 700,000 variants) to sequence. We also report on the effect of pre-filtering the reference sequence variants prior to imputation.

## Methods

### Target animal genotypes

#### Sequence genotypes

The target animals used for this study included 70 sequenced bulls: 35 Jersey (JER) and 35 Holstein (HOL). We used pedigree records and a genomic relationship matrix to confirm that there were no half-siblings present. Individuals were sequenced using Illumina HiSeq 2000 (Illumina Inc., San Diego, CA). Raw sequence reads were aligned to the ARS-UCD1.2 reference genome [26]. Alignment, variant calling, and quality controls were performed following the 1000 Bull Genomes Project guidelines as the animals were included in Run8 (See Additional 1, Text S1). In this study, we used chromosome 1, 5, 10, 15, 20, 25, and X to evaluate the accuracy of imputation at sequence level. The X chromosome was split into the non-pseudo-autosomal region (non-PAR: chromosome “30”), and the PAR (chromosome “32”). The boundary between the non-PAR and PAR was set at 133,300,518 bp [31].

#### HD and LD SNP genotypes

The same target set of 70 individuals were also genotyped using the Illumina® BovineHD 800 K bead chip (HD). The marker map positions were lifted over to the ARS-UCD1.2 reference genome using publicly available datasets (https://www.animalgenome.org/repository/cattle/UMC_bovine_coordinates/). The raw HD genotypes for these animals were processed together with the HD reference population described below. This set of HD genotypes were also masked down to a genome-wide Low Density (LD) set of 7135 SNP markers that overlap many of the commonly used current and historical SNP panels, including the Illumina® BovineSNP50K and HD SNP panels.

## Reference animal genotypes

### 50K and HD SNP genotypes

We had previously generated a reference population of 14,722 animals (representing Holstein, Jersey, and Australian Red breeds) with real lllumina^®^ BovineSNP50K panel (50K) genotypes, including a total of 40,397 SNP that passed quality filters and overlapped the Bovine Illumina HD SNP panel. Additionally, we had a cohort of 2814 animals with real HD SNP genotypes that constituted the HD imputation reference (again representing the same breeds as for the 50K reference population). The final set of HD SNP passing the quality control was 714,452 that overlapped the sequence variants in the reference population described below. In processing the raw 50K and HD genotypes, the GenCall threshold score was set at 0.6, such that SNP with a lower score were set to missing and SNP with > 10% missing genotypes were removed. All animals had < 10% missing genotypes. For both the 50K and HD reference sets, the remaining sporadic missing genotypes were imputed using FImpute v.3 [21].

#### Sequence genotypes

The imputation reference comprised 4190 taurus cattle sequences in Run8 of the 1000 Bull Genomes Project [32]. The reference sequences were processed following the 1000 Bull Genomes project pipeline (See Additional file 1, Text S1) and within this pipeline the sporadic missing genotypes were imputed using Beagle 4.1 [28]. Following imputation of the sporadic missing genotypes, variants were removed if their Beagle Rsq_soft_ was less than 0.9. The sequence variants were then further filtered to retain only bi-allelic variants with a minor allele count (MAC) of at least 4 and with a GATK [33] Variant Quality Score Recalibration (VQSR) Tranche of 99.0 or better. Additionally, we identified regions of excessive heterozygosity in sliding windows of 0.5 Mb, defined as windows where 2% or more of the variants had heterozygosity > 0.55. Within these windows, we removed all variants with heterozygosity > 0.55 because this generally indicates regions with known long segmental repeats that suffer from poor alignment of short read sequence data resulting in false SNP [34].

### Phasing and imputation strategies

We used the 70 target animals to evaluate the accuracy of imputing to sequence either directly from their real HD genotypes or starting from the LD SNP genotypes (7135 markers generated from their masked HD genotypes). The LD SNP genotypes were imputed first to the 50K reference and then up to the HD reference using FImpute v.3 with default settings and no pedigree provided. The autosomal chromosomes (Chr 1, 5, 10, 15, 20, and 25) were each imputed independently. The X chromosome non-PAR and PAR were imputed separately as per software recommendations. Finally, both the imputed and real HD genotypes of the target animals were converted to VCF format ensuring that the SNP array alleles were matched to the sequence format before final imputation to sequence level.

For sequence imputation, we evaluated the performance of three imputation tools: Beagle version 5.2 [28], IMPUTE5 version 1.1.4 [30], and Minimac4 version 1.0.2 [29]. When using IMPUTE5 and Minimac4 imputation software, genotypes of the target and reference individuals were pre-phased (as required) using Eagle v2.4.1 [35] prior to imputation. The target animal genotypes were left unphased for the Beagle imputation because this software does not require pre-phasing. We ran a preliminary investigation of the accuracy of Beagle imputation using either the Eagle-phased reference, or a Beagle-phased reference. Our analysis found little difference in imputation accuracy between Eagle- or Beagle-phasing of the reference, although Eagle-phased reference resulted in slightly higher accuracy (0.4% on average) in both Best-Guess (GT) and Dosage (DS) genotypes (See Additional file 2, Table S1, Sheet 1). We therefore decided to use the Eagle-phased reference for all software benchmarking to maintain consistency across scenarios.

**Fig. 1:**
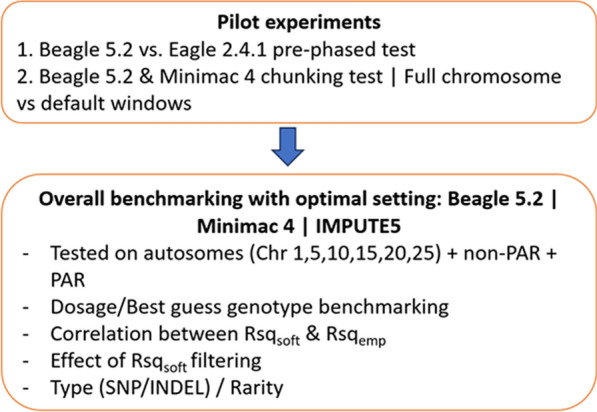
Flowchart showing overall experimental design for empirical testing of imputation accuracy and benchmarking the software estimate of imputation accuracy

Additionally, as a pilot investigation, we compared the empirical accuracy of sequence imputation for Beagle 5.2 and Minimac4 using two different settings relating to the length of haplotype imputed. As a default setting for computational efficiency, both programs implement an automated “chunking” of chromosomes into shorter window lengths. Conversely, the default setting in IMPUTE5 is to use the full chromosome. The Beagle 5.2 default sets the window lengths to 40 centiMorgan (with a 2 cM overlap) while the Minimac4 default is 20 Mb with a 3 Mb overlap. We therefore tested Beagle 5.2 and Minimac4 using either the default window size or full chromosome lengths under the hypothesis that use of the full chromosome might improve imputation accuracy by taking advantage of the long-distance linkage disequilibrium in cattle. Figure 1 illustrates the overall experimental design of the study.

The settings tested were:

#### Beagle 5.2

1. D (Default setting): window = 40.0, overlap = 2.0 (distances are in cM) or
2. F (Full chromosome): window = 200.0, overlap = 80.0 (ensured that one ‘window’ is larger than the entire length of each chromosome)

For both tests above, a seed setting was used to ensure reproducibility (seed value = 5555). The effective population size parameter (*Ne*) was set at 1000 for all Beagle imputation.

#### Minimac4

1. D (Default setting): –ChunkLengthMb 20 –ChunkOverlapMb 3 or
2. F (Full chromosome): –ChunkLengthMb 200 –ChunkOverlapMb 80 (ensured that one ‘window’ was larger than the entire length of each chromosome).

No seed or effective population size setting was available in Minimac4.

#### IMPUTE5

The default settings of IMPUTE5 were used, except that *Ne* was set at 1000. No seed setting was available in IMPUTE5.

The results from the pre-phasing and pilot test described above were used to inform the settings used in the main benchmarking study of the three software programs.

### Imputation accuracy statistics

We defined *empirical accuracy* (*r*) as Pearson correlation coefficient between the real sequence genotypes (coded as the number of alternate alleles: 0, 1, or 2) and imputed genotypes. The imputed genotypes were available in two forms: either allele “dosage” (DS: continuous measures between 0 to 2, representing the sum of the two alternate allele probabilities) or “best-guess” genotypes (GT: coded with 0, 1 or 2 as for real genotypes). The correlation was calculated for both DS and GT on a per variant basis across all target animals.

A variant had to be segregating in both the real and imputed best guess genotypes (GT) of the target set in order to calculate *r*, otherwise the variance for that position is zero and *r* cannot be estimated. For DS genotypes, there is always some variance when the variant is segregating in the real genotypes of the target set. Therefore, unless stated otherwise, when benchmarking across different software, we compared only the common set of overlapping variants that had an estimable *r* across all software for GT genotypes. Additionally, we use the DS imputed genotypes to enable comparisons across the entire set of variants segregating in the target set. An internal software estimate of imputation accuracy per variant was available in the output of all three packages (we will refer to as: Rsq_soft_) and this was compared to the squared empirical accuracy of imputation (Rsq_emp_).

For imputed GT genotypes, we measured three additional, more specific statistics:

1. *False positive error rate* (FPR): defined as the average percentage of reference alleles that were wrongly imputed as the alternate allele for any given variant position.
2. *False negative error rate* (FNR): defined as the average percentage of alternate alleles that are wrongly imputed as the reference allele for any given variant position.
3. *Allelic imputation error rate* (AER): defined as the average percentage of alleles that are wrongly imputed (i.e. false positives + false negatives) for any given variant position.

A graphical schematic on the calculations of these statistics can be found in Additional file 3, Figure S1.

## Results

### Chromosome chunking versus full chromosome imputation (Beagle 5.2 and Minimac4)

The default settings in both Minimac4 and Beagle 5.2 divide chromosomes into smaller overlapping windows as a means of speeding up the imputation process while the default for IMPUTE5 is to use full length chromosomes. Therefore, for Beagle 5.2 and Minimac4 we compared their default window settings (D) with imputing the full chromosome (F) as a single window. We tested this because cattle have a small recent effective population size [36] and often the target and reference animals are quite closely related so may share long haplotypes. However, our results from the different parameter settings (D versus F) did not show significantly different imputation accuracy (See Additional file 2, Table S1, Sheet 2). We therefore proceeded with the default window settings for Minimac4 & Beagle 5.2 for the remainder of the study comparisons.

### Empirical imputation accuracy

**Fig. 2:**
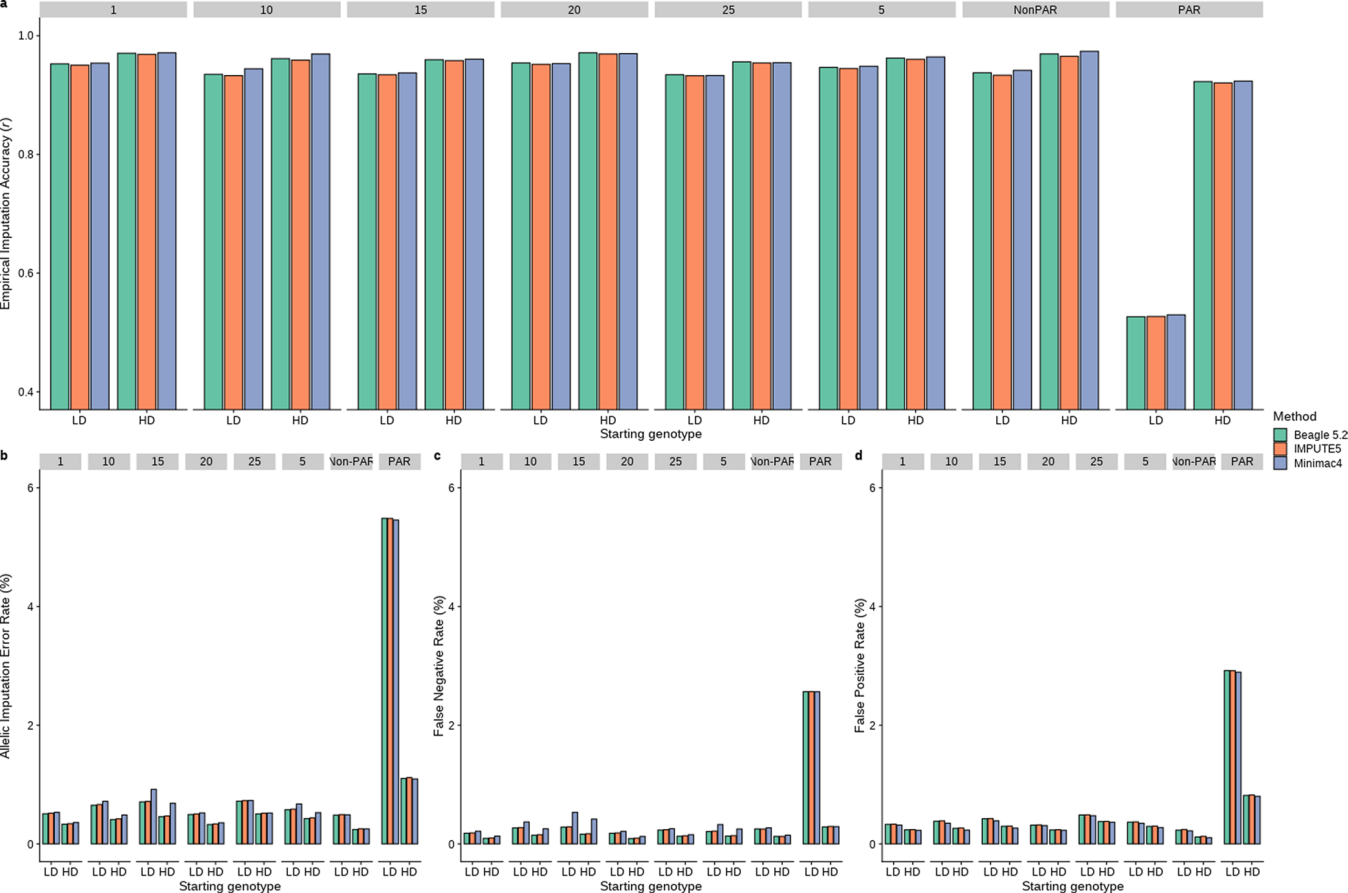
Imputation statistics for six autosomes (1, 5, 10, 15, 20 & 25) as well as the X chromosome non-pseudo-autosomal region (*non-PAR*) and pseudo-autosomal region (*PAR*) for three imputation software (Beagle 5.2, Minimac4 and IMPUTE5). **a** Average empirical imputation accuracy (r) for best guess genotypes (GT) and considering all variants imputed as segregating by the specific software. **b** Average allelic imputation error rate (%). **c** False Negative Rate (% of alternate alleles wrongly imputed as reference). **d** False Positive Rate (% of reference alleles wrongly imputed as alternate). HD = Impute from real HD genotypes to sequence. LD = Impute from low density to 50K to HD to sequence

For all three software methods, we first compared the *r* of imputation to sequence starting either direct from real HD genotypes, or from LD genotypes (approximately 7K SNP) imputed to 50K, then to HD and finally to sequence. For all autosomes tested (1, 5, 10, 15, 25) as well as the non-PAR of the X chromosome, there was a small (~ 2%) but consistent drop in accuracy of best guess genotypes (GT) when imputing from LD genotypes compared to starting from real HD genotypes (Fig. 2a). However, for the PAR region of the X chromosome there was a very sharp drop in *r* from around 0.9 (starting from HD) to less than 0.6 when starting from LD genotypes (Fig. 2a). Overall, there were only small differences in *r* across the autosomal chromosomes and the non-PAR (Fig. 2a).

There was almost no difference between software in the accuracy of imputation measured as the correlation of best guess (GT) genotypes (Fig. 2a). Likewise, the concordance rate for best guess genotypes (GT mode) was relatively high across all programs tested, ranging from 90.0 to 99.0% (imputing from LD) and 97.8% to 99.6% for imputing from real HD (See Additional file 2, Table S2, Sheet 3). However, *r* in Fig. 2 included all variants that the given program imputed as segregating, so when comparing software performance, this result can show some bias if a specific software has a stronger tendency to impute rare variants as not segregating because the correlation is not estimable for those variants. Therefore, we also estimated AER (allelic error rate), FNR (alternate alleles falsely imputed as reference alleles), and FPR (reference alleles falsely imputed as alternate alleles) statistics. Minimac4 tended to produce a slightly higher AER and FNR compared to either Beagle 5.2 or IMPUTE5, while the FPR was relatively stable across software (Fig. 2b–d). Thus, the overall number of variants for which the correlation was estimable was approximately 3% lower for Minimac4 compared to that of Beagle 5.2 and IMPUTE5 (starting from LD: 3,961,994 versus 4,086,017 and 4,076,376 variants and starting from HD: 3,992,240 versus 4,119,862 and 4,109,502, respectively). We also estimated the percentage of segregating variants that were imputed as not segregating (Chromosome 1, starting from HD) and found that the highest error rate was for Minimac4 (3.5%) compared to Beagle 5.2 and IMPUTE5 (1.5% and 1.8%, respectively). Conversely, the percentage error rate for non-segregating variants imputed as segregating was low across all software (0.3% for Minimac4, 0.7% of Beagle 5.2, and 0.8% of IMPUTE5). As expected, the AER, FPR and FNR were always higher when imputation started from LD rather than from real HD genotypes. Additionally, while there were only small differences between the autosomal chromosomes, the AER, FPR and FNR were much higher for the PAR of chromosome X (Fig. 2b–d).

Additionally, we re-estimated the average *r* within only the overlapping set of variants (DS and GT) that had an estimable *r* across all three software programs (i.e., removing all variants imputed as monomorphic by any software). For DS genotypes we show the correlation for this overlapping variant set as well as for the full variant set because with DS most variants show some small imputation probability of segregating. We combined all the autosomal variants for this comparison and Table 1 shows the *r* using either GT or DS genotypes for variants from the autosomes, PAR and Non-PAR. All programs performed similarly, and as expected, the correlation was slightly higher for DS genotypes compared to GT genotypes. The DS and GT accuracies for the non-PAR were similar to the autosomes across all comparisons, while the PAR had a very low accuracy of imputation when starting from LD genotypes. The accuracy of imputation on the PAR starting from real HD genotypes was much higher than when starting from LD but was still ~ 2 to 4% lower than for the autosomes.

**Table 1:** Average empirical imputation accuracy (*r*) per variant when imputing from either low (LD) or high density (HD) genotypes using three imputation programs (Beagle 5.2, Minimac4 and IMPUTE5)

		Autosome (*r* ± SD)		Non-PAR (*r* ± SD)		PAR (*r* ± SD)	
Mode	Programs	LD	HD	LD	HD	LD	HD
GT^1^	Beagle 5.2	0.943 ± 0.114	0.964 ± 0.101	0.938 ± 0.141	0.969 ± 0.128	0.527 ± 0.196	0.923 ± 0.105
	Minimac4	0.945 ± 0.101	0.965 ± 0.088	0.942 ± 0.13	0.974 ± 0.113	0.53 ± 0.2	0.923 ± 0.099
	IMPUTE5	0.941 ± 0.116	0.962 ± 0.103	0.934 ± 0.153	0.965 ± 0.14	0.53 ± 0.2	0.92 ± 0.107
DS^1^ (Same variant set as GT)	Beagle 5.2	0.946 ± 0.111	0.966 ± 0.097	0.941 ± 0.134	0.972 ± 0.12	0.53 ± 0.2	0.925 ± 0.101
	Minimac4	0.95 ± 0.09	0.969 ± 0.073	0.945 ± 0.122	0.977 ± 0.104	0.53 ± 0.19	0.926 ± 0.089
	IMPUTE5	0.945 ± 0.112	0.965 ± 0.098	0.936 ± 0.148	0.968 ± 0.134	0.53 ± 0.2	0.923 ± 0.102
DS^1^ (All variant set)	Beagle 5.2	0.938 ± 0.133	0.96 ± 0.119	0.907 ± 0.219	0.941 ± 0.209	0.52 ± 0.2	0.92 ± 0.121
	Minimac4	0.922 ± 0.169	0.946 ± 0.15	0.893 ± 0.243	0.927 ± 0.232	0.51 ± 0.22	0.914 ± 0.134
	IMPUTE5	0.935 ± 0.142	0.956 ± 0.128	0.909 ± 0.215	0.942 ± 0.206	0.52 ± 0.2	0.918 ± 0.123

Accuracy is shown separately for autosomal chromosomes (Autosome: Chr 1, 5, 10, 15, 20, 25), the non-PAR and PAR of the X chromosome

^1^Accuracy was calculated as the correlation between imputed and real genotypes, where imputed genotypes were either best guess (GT) or allele dosage (DS). The accuracy for GT was calculated only from the union of variants that were imputed as segregating across all three software tools, while DS accuracy is shown for both the GT union set as well as for all variants segregating in the real genotype data

Within the chromosomes tested, there was a total of 748,461 INDEL representing 7% of all the imputed variants in our study. We compared the accuracy of imputation between SNP and INDEL for DS genotypes across all tested autosomes as well as the PAR and non-PAR, imputing either from real HD or from LD to sequence, using the three software tools (Table 2). On average, for all software programs the INDEL had approximately 6%, 14% and 4% lower imputation accuracy compared to SNP for autosomes, non-PAR and PAR respectively. However, for autosomal variants the Beagle 5.2 INDEL imputation accuracy was slightly better than IMPUTE5 and in all comparisons, Beagle accuracy was nearly 2% better than Minimac4 when imputing from LD.

**Table 2:** Comparison of imputation accuracy (correlation between real and imputed allele dosage genotypes) for all imputed SNP and INDEL, using Beagle 5.2, Minimac4, or IMPUTE5

	Autosome (Number of Variants = 8,890,108)				Non-PAR (Number of Variants = 763,172)				PAR (Number of Variants = 143,831)			
	LD		HD		LD		HD		LD		HD	
	INDEL	SNP	INDEL	SNP	INDEL	SNP	INDEL	SNP	INDEL	SNP	INDEL	SNP
Beagle 5.2	0.892	0.944	0.912	0.965	0.780	0.920	0.813	0.954	0.481	0.525	0.857	0.925
Minimac4	0.867	0.928	0.888	0.952	0.752	0.908	0.784	0.943	0.472	0.517	0.843	0.920
IMPUTE5	0.884	0.941	0.904	0.962	0.785	0.922	0.816	0.955	0.483	0.525	0.855	0.923

Variants were imputed either direct from real HD (high density) or from LD (low density) genotypes. The accuracies are shown separately for autosomes (Chr 1, 5, 10, 15, 20, 25), the non-PAR and the PAR of the X chromosome

### Software imputation quality metric versus empirical accuracy

**Fig. 3:**
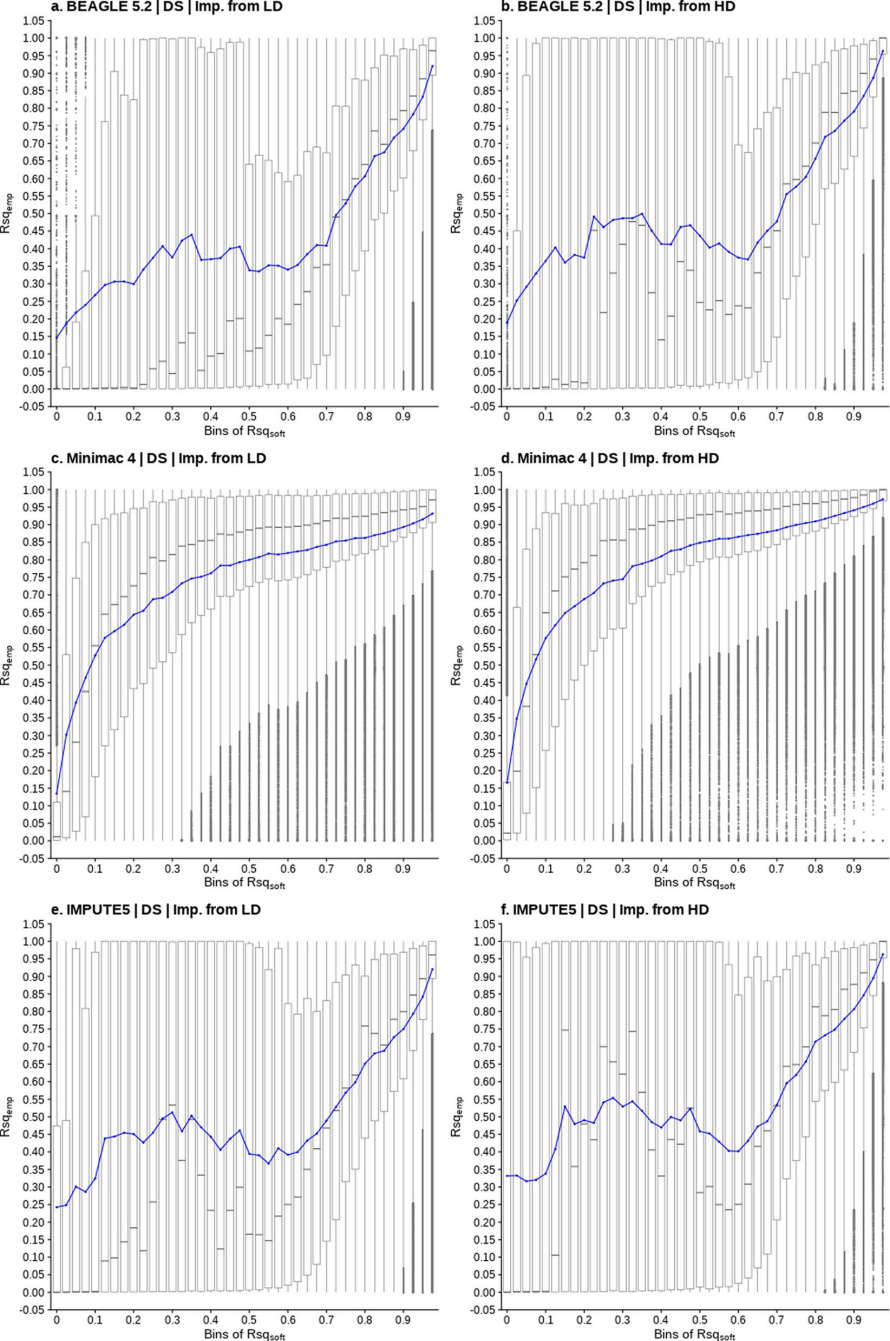
Boxplots showing the relationship between bins of Rsq_soft_ (x-axis) and the distribution of Rsq_emp_ (y-axis) for imputed dosage (DS) genotypes on all chromosomes tested. The blue line represents the average Rsq_emp_ value at each bin and the grey line within each box represents the median value. The box contains the 25th to the 75th percentile of the data points. Whiskers extend to a maximum length of 1.5 times the interquartile range (IQR) beyond the box edges. Data points beyond the whiskers are represented by individual dots as outliers. Plots (**a**), **c** and **e** show results for imputation starting from Low-density genotypes (LD: Beagle 5.2, Minimac4 and IMPUTE5) while plots (**b**), **d** and **f** show results for imputation starting from high-density genotypes (HD: Beagle 5.2, Minimac4 and IMPUTE5)

After establishing the accuracy of imputation from LD and HD genotypes to sequence level, this part of the study addresses two critical questions. First, is the relationship between the software estimated accuracy (Rsq_soft_) and empirical imputation accuracy (Rsq_emp_) strong enough to provide a useful means of filtering poorly imputed data? Second, what is an appropriate imputation Rsq_soft_ threshold for each software? The relationship between the software imputation quality measure (Rsq_soft_) and the empirical accuracy as the squared correlation (Rsq_emp_) is shown in Fig. 3, when imputing from either LD or HD, and using DS mode for all three imputation programs. The results are provided for variants on all chromosomes (1, 5, 10, 15, 20, 25, non-PAR and PAR) and the equivalent results with GT mode are also available (See Additional file 4, Figure S2). There was a strong relationship between Rsq_soft_ and Rsq_emp_ although it is not linear and is very different for Minimac4 compared to Beagle 5.2 and IMPUTE5. For all imputation tools, Rsq_soft_ at higher values shows a closer relationship with Rsq_emp_. The boxplot distributions show considerable variation of Rsq_emp_ within the bins of Rsq_soft_ at low to mid-range values, particularly for Beagle and IMPUTE5. This is partly a function of there being fewer variants in these Rsq_soft_ bins. Conversely, at the higher Rsq_soft_ values that fall within the useful range for filtering, the boxplots become less dispersed indicating that the relationship between Rsq_soft_ and Rsq_emp_ is more reliable.

The results in Fig. 3 allow an equivalent Rsq_soft_ threshold to be identified for each software that could be employed to filter imputed data for downstream analysis. For example, an Rsq_soft_ threshold of ~ 0.4 in Minimac4 and ~ 0.9 for Beagle 5.2 and IMPUTE5 would remove sequence variants with an average Rsq_emp_ lower than ~ 0.8 (*r* = 0.89) for data imputed from HD genotypes.

**Fig. 4:**
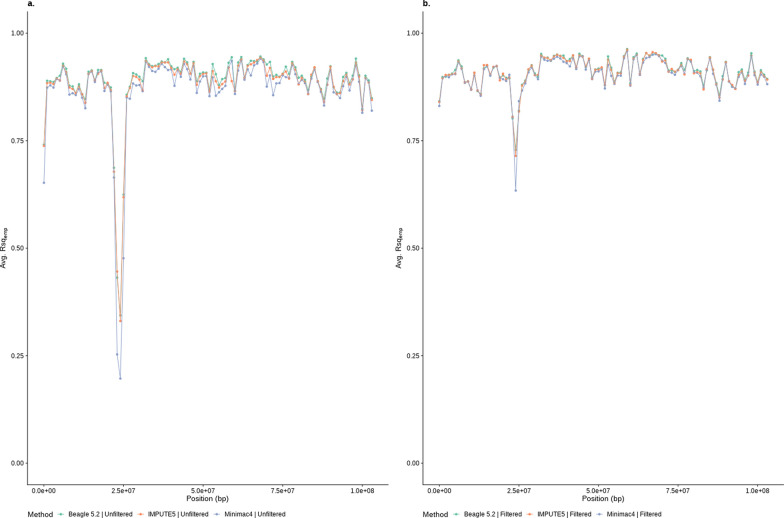
Average imputation accuracy (Rsq_emp_) for all variants in each adjacent 1 Mb window across chromosome 10. Using either no Rsq_soft_ filter (**a**) or after imposing an Rsq_soft_ threshold filter (**b**) for three different imputation programs (Rsq_soft_ threshold > 0.4 for Minimac4 and > 0.9 for Beagle 5.2 and IMPUTE5). Variants imputed from low density for imputed dosage (DS) genotypes

It is known that some chromosome regions are difficult to accurately impute [24, 25], therefore it is also of interest to understand if use of an Rsq_Soft_ filter will accurately remove poorly imputed variants and thus improve the average Rsq_emp_ across these regions. Figure 4a shows Rsq_emp_ averaged across all variants within windows of 1 Mb (Chromosome 10) with no filter on Rsq_Soft_ compared to Fig. 4b where the variants were first filtered using an Rsq_Soft_ threshold (0.4 for Minimac4 and 0.9 for Beagle 5.2 and IMPUTE5). Filtering improved the average Rsq_emp_ across this entire chromosome but had a particularly large impact across a 4 Mb segment that showed very poor average Rsq_emp_ before filtering, as well as at the ends of the chromosome. The filtering evened out any differences between software in the distribution of the Rsq_emp_ across the chromosome except at the very poorly imputed region where Minimac4 still showed a lower Rsq_emp_.

Clearly, the Rsq_soft_ threshold is a useful indicator of Rsq_emp_, but it is also important to quantify the resulting proportion of false positives (FP: variants that passed the Rsq_soft_ threshold but did not reach the expected Rsq_emp_) and false negatives (FN: variants that did not pass the Rsq_soft_ threshold but did achieve the desired Rsq_emp_). The FP and FN results are shown in Table 3 after imposing the equivalent Rsq_soft_ thresholds (specific to Minimac4, Beagle and IMPUTE5) as demonstrated in Fig. 3 to achieve an average Rsq_emp_ ≥ 0.8 (imputing from real HD genotypes and using DS genotypes). The proportion of variants passing the equivalent Rsq_soft_ thresholds was similar for all three imputation programs as was the proportion of false negatives and false positives, although Beagle 5.2 tended to show the lowest level of false negatives/positives.

**Table 3:** Comparison of variant filtering using equivalent Rsq_soft_ thresholds from three imputation tools (Beagle 5.2, Minimac4 and IMPUTE5) to achieve an empirical accuracy (Rsq_emp_) > 0.8

Imputation Software	Total No. of variants passing Rsq_soft_ (% of all variants imputed)	True positives: No. variants passing Rsq_soft_ & Rsq_emp_ (expressed as % of variants passing Rsq_soft_)	False positives: No. variants passing Rsq_soft_ & failing Rsq_emp_ (expressed as % of variants passing Rsq_soft_)	False negatives: No. variants not passing Rsq_soft_ but passing expected Rsq_emp_ (expressed as % of variants not passing the Rsq_soft_)
Beagle 5.2	3,991,626 (40.7%)	3,748,177 (93.9%)	243,449 (6.1%)	66,135 (1.1%)
IMPUTE5	3,954,595 (40.4%)	3,702,798 (93.6%)	251,797 (6.4%)	101,575 (1.7%)
Minimac4	3,949,886 (40.3%)	3,679,117 (93.1%)	270,769 (6.9%)	88,715 (1.5%)

The Rsq_soft_ thresholds were 0.4 for Minimac4, 0.9 for Beagle 5.2 and 0.9 for IMPUTE5 and were applied to dosage genotypes imputed from real HD genotypes

**Fig. 5:**
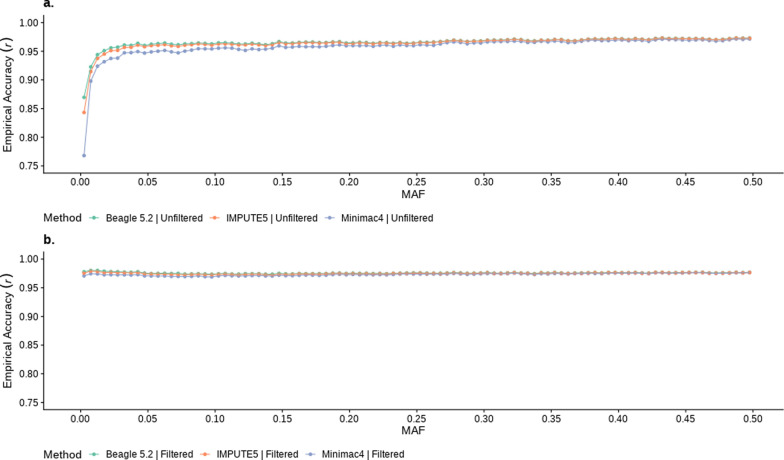
Average imputation accuracy (Rsq_emp_) for sequence variants binned by minor allele frequency (MAF) using three different imputation programs (Beagle 5.2, Minimac4 and IMPUTE5). (**a**) shows Rsq_emp_ without imposing any Rsq_soft_ filter, while (**b**) is Rsq_emp_ after imposing an Rsq_soft_ filter (Rsq_soft_ > 0.4 for Minimac4, > 0.9 for Beagle 5.2 and IMPUTE5). Imputation started from real HD genotypes and accuracy was assessed for dosage genotypes

We also investigated the effect of Rsq_soft_ filtering on empirical imputation accuracy for all variants grouped on minor allele frequency (MAF: Fig. 5) in the imputation reference population. As expected, with no Rsq_soft_ filter, the lower MAF variant bins show considerably lower empirical accuracy compared to MAF > 0.025 (Fig. 5a). Conversely, after applying an Rsq_soft_ filter (> 0.4 for Minimac4, and > 0.9 for Beagle & IMPUTE5), the average empirical accuracy for the remaining low MAF variants was significantly increased and very close to those with higher MAF (Fig. 5b).

We then divided all imputed variants into two groups: either common (MAF > 0.05) or less common (MAF < 0.05) and compared the effect of applying an Rsq_soft_ threshold on the empirical imputation accuracy of INDEL and SNP (Table 4). Generally, the Rsq_soft_ filter for all software removed a similar proportion of variants for either SNP or INDEL. However, the proportion of common variants (MAF > 0.05) passing the Rsq_soft_ filters was very high (> 93%) while only ~ 16% of less common variants passed the filters. One interesting phenomenon we observed is that the false positive rate of INDEL was always higher than that of SNP (for both common and less common MAF) implying that the INDEL empirical imputation accuracy is less well predicted by the Rsq_soft_ filter than it is for SNP.

**Table 4:** Comparison of variant filtering using equivalent Rsq_soft_ thresholds from three imputation tools (Beagle 5.2, Minimac4 and IMPUTE5), separated by SNP and INDEL variants with MAF either above or below 0.05

		SNP			INDEL		
	Imputation Software	Percent of variants passing Rsqsoft (Number of variant)	False positives: Percent of variants passing Rsq_soft_ but not Rsq_emp_ threshold	False negatives: Percent of variants not passing Rsq_soft_ but passing Rsq_emp_ threshold	Percent of variants passing Rsq_soft_ (Number of variant)	False positives: Percent of variants passing Rsq_soft_ but not Rsq_emp_ threshold	False negatives: Percent of variants not passing Rsq_soft_ but passing Rsq_emp_ threshold
Common variants (MAF > 0.05 in Reference population)	Beagle 5.2	95.82 (2,671,196)	4.80	0.52	93.54 (230,666)	6.31	0.55
	Minimac4	95.08 (2,663,244)	5.20	0.84	93.47 (230,494)	7.06	0.81
	IMPUTE5	95.53 (2,650,696)	4.95	0.77	93.33 (230,135)	6.49	0.77
Less common Variants (MAF < 0.05 in Reference population)	Beagle 5.2	16.07 (1,006,321)	1.31	0.73	16.63 (83,443)	2.35	0.87
	Minimac4	15.73 (985,045)	1.51	0.93	15.97 (80,127)	2.74	0.96
	IMPUTE5	15.64 (979,105)	1.38	1.15	16.10 (80,807)	2.32	1.28

Variants were imputed from real HD genotypes and accuracy was assessed using dosage genotypes. The Rsq_soft_ thresholds were chosen for each software to achieve an Rsq_emp_ of ≥ 0.8 (Rsq_soft_ of 0.4 for Minimac4, 0.9 for Beagle 5.2 and 0.9 for IMPUTE5). The percentage of variants passing the Rsq_soft_ filter is shown for each of the variant categories, as well as the percentage of false positives and negatives

## Discussion

This study provides a comprehensive evaluation of empirical imputation accuracy of sequence variants using three popular and computationally efficient imputation programs that provide an internal quality statistic of imputation accuracy (Rsq_soft_). This is a unique study evaluating the empirical accuracy of imputing LD (7K) and HD genotypes to sequence: providing direct comparisons of accuracy for the PAR, non-PAR and autosomes, as well as INDEL and SNP variants. An additional novelty of this study was an in-depth evaluation of the relationship between Rsq_soft_ and Rsq_emp_ for the three imputation programs, as well as an assessment of the value of in-house pre-filtering of sequence variants in the reference population.

We demonstrated that the Rsq_soft_ filtering thresholds were similar for Beagle 5.2 and IMPUTE5 but differed for Minimac4. While each tool employs a different Rsq_soft_ algorithm, the Beagle 5.2 and IMPUTE5 Rsq_soft_ have previously been shown to be strongly correlated for the same imputation sample [1]. Beagle estimates the squared correlation between the imputed genotypes and true genotypes, where the (co)variance of the true genotypes is approximated using the sample mean of imputed genotypes [28, 37]. The IMPUTE5 algorithm measures the ratio of observed and complete information by considering the relative statistical information about the population allele frequency [1]. Minimac4 algorithm computes the average squared deviation of the imputed allele dosage at each haplotype in the sample relative to the estimated allele frequency, and divides this by the product of the alternate and reference allele frequency [38]. Despite these differences, our empirical tests demonstrated that if an appropriate Rsq_soft_ filter was applied, the majority of poorly imputed variants were removed, with those remaining having a higher and similar average empirical imputation accuracy. Importantly, Fig. 3 provides an empirical determination of the equivalent Rsq_soft_ thresholds across software that would maintain a common baseline Rsq_emp_ for downstream analyses (such as meta-GWAS). Interestingly, the Rsq_soft_ of both Beagle 5.2 and IMPUTE5 shows an overprediction of the Rsq_emp_ for the higher Rsq_soft_ values while the reverse is true of Minimac4. A previous study using Minimac3 to impute sequence variants in sheep [39] reported a very similar relationship to that found in our study and a study in chickens also reported that Beagle Rsq_soft_ overpredicted the Rsq_emp_ [40]. We also demonstrated that in practice, the application of the equivalent thresholds from the three imputation tools resulted in similar numbers of variants being discarded and reasonably numbers of low false positives and negatives. A recent study has attempted to address the variability of Rsq_soft_ by developing a machine learning based quality calibration measure [41], but a drawback of that approach is that the model must first be trained on real, high quality genotypes at a range of frequencies.

Typically, for large-scale sequence imputation the software can parallelise the workload by splitting chromosomes into smaller segments (‘chunking’) which can dramatically speed up the imputation time required but may incur a penalty on accuracy. Several studies have documented the trade-off between accuracy and computational efficiency of imputation programs previously [30, 42–44]. We were concerned that the default chunking in Minimac4 and Beagle 5.2 might significantly reduce imputation accuracy because cattle breeds have small effective population sizes that can result in long haplotype blocks from extended regions of high linkage disequilibrium [45]. Interestingly, we found no advantage in imputation accuracy when the default setting was modified to allow entire chromosome imputation without chunking. This may partly relate to the current limitations of accurately defining longer haplotypes within short-read sequence [46]. We did not explore reducing chunk size settings below the default settings because these tools were primarily developed and tested in human data where the effective population size is generally much larger than in cattle.

We did not benchmark the computational efficiency of the software because a comprehensive study with human data has been published [30] where it was shown that the relative efficiencies between software changed with the size of the reference population. Using a modest sized reference population (N = 2504) and chunk size of 20 Mb for all software, Minimac4 imputation was found to be considerable slower than IMPUTE5 and Beagle v5.1, while IMPUTE5 used the least memory [30]. This suggests that if imputation time is a concern, it may be preferable to use either Beagle 5.2 or IMPUTE5 rather than using Minimac4. An additional study with human data benchmarked computational efficiency of the same software based on their default settings (no chunking in IMPUTE5, 20 Mb chunks in Minimac4 and 40 Mb in Beagle 5.2) and including phasing time [42]. They reported that Beagle 5.4 (with Beagle 5.4 phasing) was considerably faster than IMPUTE5 or Minimac4 when both the latter were pre-phased with Eagle 2.4.1.

As expected, imputed allele dosage genotypes were more highly correlated to the real genotypes than imputed best-guess genotypes and previous literature has shown that there may be a small benefit in using allele dosage versus best guess genotypes for GWAS [6]. However, there is an increased computational burden incurred for analyses that use dosage genotypes rather than best guess genotypes.

Our finding of reduced imputation accuracy for low MAF variants agrees with several previous studies where a larger multibreed reference helped to improve the accuracy of low MAF variants compared to a single breed [24]. However, only one previous study in cattle reported the accuracy of imputing sequence INDEL compared to SNP, and as in our study they reported that INDEL were less well imputed than SNP [47]. In general, given that INDEL can vary in length and complexity, and may overlap SNP sites, it is plausible that they are more difficult to accurately detect and impute. In addition, it is possible that a higher proportion of segregating INDEL tend to be more recent mutations than SNP, being more likely to be functionally disruptive and under stronger purging selection. Recent mutations may be harder to impute accurately compared to older mutations because some haplotypes surrounding the mutation may still be segregating in the reference population without the mutation [48]

We found no other cattle studies that have specifically considered the imputation accuracy of sequence variants from SNP chip to sequence on the PAR and non-PAR of the X chromosome. Two previous studies reported accuracy of imputation from LD to 50K SNP density for autosomes, the non-PAR and PAR [49, 50] using the previous cattle reference genome (UMD3.1) and reported much lower imputation accuracy for the PAR. Both those studies suggested that the low accuracy may be partly attributed to the short length of the PAR (5.7 Mb). We tested this hypothesis in a recent study [51] by comparing the PAR imputation accuracy with the accuracy of imputing short 5.7 Mb autosomal segments that were extracted from the ends of five different chromosomes (with SNP density was adjusted to match). These autosomal segments were imputed from LD genotypes to 50K and HD genotypes and although their imputation accuracy was lower than for whole autosomal chromosomes, it was still considerably higher than for the PAR [51]. Furthermore, in the current study we demonstrated that when imputing from real HD genotypes to sequence, the PAR imputation recovered considerable accuracy (Table 1). This suggests that the low imputation accuracy for LD genotypes on the PAR is likely due to the higher recombination rate of the PAR [52, 53] and is therefore a generalised result that would be observed across cattle populations. Although the PAR represents only 4% of the X chromosome, it would be useful to increase the density of PAR markers compared to other chromosomes when generating custom SNP panels, because this could significantly improve the PAR imputation accuracy.

In contrast to the PAR, our study showed that the imputation accuracy on the non-PAR was similar to the autosomes. This is important because many cattle studies that use imputed sequence in downstream studies such as GWAS, have discarded the entire X chromosome before undertaking downstream analyses, suggesting a lost opportunity (e.g. [54]). Our target animals were males, and potentially because males are haploid across the non-PAR (presented as diploid to the imputation software), they may be a little more accurately phased than diploid females. If phasing is a little less accurate in target females this could lead to a lower imputation accuracy than for males, but this has not been tested empirically because we did not have equivalent female samples.

## Conclusions

Our findings offer valuable insights on the application of imputation filters across software for downstream analyses such as meta-GWAS studies. It is likely that these imputation tools will remain popular because they showed similarly high imputation accuracy. This study demonstrates that Rsq_soft_ is a useful filtering tool for both SNP and INDEL. We provide a generalised empirical determination for equivalent Rsq_soft_ thresholds across the three imputation tools. The extremely low accuracy observed for imputation from LD to sequence on the PAR of the X chromosome indicates that imputed data in this region cannot be confidently used for downstream analyses.

## Supplementary Information

Additional file 1: Protocol to process sequence data in Run8 of the 1000 Bull Genomes Project [27, 55, 56]. Text documentation describing the pipeline and software versions used to process sequence data.

Additional file 2: Detail statistics of the analysis. Excel file containing three sheets. Sheet 1 - Statistics on Phasing result between Dosage (DS) vs Genotype likelihoods (GT) mode. Sheet 2 – Correlation statistics between different mode of running of Beagle and MINIMAC4. Sheet 3 – Correlation statistics when imputing from LD or HD across 3 programs Beagle 5.2, MINIMAC4 and IMPUTE5.

Additional file 3: Graphical schematics of imputation cases used in the current analysis. REF represents the reference allele, ALT represents the alternative allele.

Additional file 4: Boxplot visualizing relationship between Rsq_soft_ (Dosage R-squared - DR2) and Rsq_emp_ (Empirical Correlation Squared) using imputed Genotype likelihoods (GT) on all chromosome tested using 3 programs Beagle 5.2, MINIMAC4 and IMPUTE5. The box contains the 25th to the 75th percentile of the data points. Whiskers extend to a maximum length of 1.5 times the interquartile range (IQR) beyond the box edges. Data points beyond the whiskers are represented by individual dots as outliers. (optional).

## Data Availability

All public sequence data from Run8 and Run9 of the 1000 Bull Genomes Project are accessible with the accession number PRJEB42783 and PRJEB56689, respectively.
