# Supplementary material for: Empirical versus estimated accuracy of imputation: optimising filtering thresholds for sequence imputation
Source: Genet Sel Evol. 2024 Nov 15;56:72. doi: 10.1186/s12711-024-00942-2 (PMC11566673; doi:10.1186/s12711-024-00942-2)
Supplement: Supplementary file 1 — Additional file 1: Protocol to process sequence data in Run8 of the 1000 Bull Genomes Project [27, 55, 56]. Text documentation describing the pipeline and software versions used to process sequence data. [file 12711_2024_942_MOESM1_ESM.pdf]

Run 8 1000 bulls GATK fastq to GVCF guidelines (GATKv3.8)

Version: 17/10/2019

These specifications describe the software and steps to process fastq files into bam and GVCF files for the 1000 Bull Genomes Project.

Data accepted

Data accepted was only from ILLUMINA sequencers, not from PacBio or Oxford Nanopore instruments at this time.

Required software

- Trimmomatic 0.38 (http://www.usadellab.org/cms/?page=trimmomatic)
    - You may use other software for trimming and quality control as long as our standards are followed
- FastQC 11.7 (http://www.bioinformatics.babraham.ac.uk/projects/download.html#fastqc)
- BWA 0.7.17 (https://github.com/lh3/bwa)
- Samtools 1.8 (http://www.htslib.org/download/)
- Tabix 1.8 (http://www.htslib.org/download/)
- Picard v2.18.2 (http://broadinstitute.github.io/picard/)
    - Requires Java 1.8 be installed
- GATK 3.8-1-0-gf15c1c3ef (there are two GATK3.8 versions, this exact version must be used) (https://software.broadinstitute.org/gatk/download/auth?package=GATK-archive&version=3.8-1-0-gf15c1c3ef)
    - Requires Java 1.8 be installed

NOTE: It is important to use GATK3.8 for all GATK steps as pointed out by GATK developers (https://gatkforums.broadinstitute.org/gatk/discussion/3536/can-i-use-different-versions-of-the-gatk-at-different-steps-of-my-analysis). If you do not use this exact GATK version, GATK will not allow us to combine your bam or GVCFs with the other project data when running GenotypeGVCFs, as versions will be inconsistent.

Starting from raw fastq versus bam file extracted fastq

We recommend that partners process their sequence starting from raw fastq format. If you do extract reads from a bam file you can do so with Picard SamToFastq tool (https://broadinstitute.github.io/picard/command-line-overview.html#SamToFastq), though other tools are available. This tool can output reads based on read groups, so if read groups are specified correctly (see https://gatkforums.broadinstitute.org/gatk/discussion/6472/read-groups for definitions) then this will re-create the original fastq files. Please make sure the original per base quality score (OQ) is associated with reads, Picards RevertSam tool (https://software.broadinstitute.org/gatk/documentation/tooldocs/4.beta.6/picard_sam_RevertSam.php) can revert previously recalibrated qscores. If you don't know the quality control that was used on the read data in the bam file and you have raw fastq files available, please start from fastq as per our guidelines in this document.

OFFICIAL

An example command for picard SamToFastq

```
java -XX:ParallelGCThreads=12 -Xmx300G -jar picard.jar SamToFastq INPUT=${InputBam}
OUTPUT_PER_RG=true COMPRESS_OUTPUTS_PER_RG=true OUTPUT_DIR=${FastqDir} RG_TAG=ID
VALIDATION_STRINGENCY=LENIENT
```

NOTE: this is for a BAM file containing read groups.

Trim and filter fastq

Trim paired reads of adapter, low quality bases (qscore <20) at the beginning and end, then filter out reads with mean qscore less than 20 or length less than 35bp. We recommend Trimmomatic because it is well documented and actively maintained. However there are many programs capable of performing this task, ie qualityTrim (https://bitbucket.org/arobinson/qualitytrim), fastp (https://github.com/OpenGene/fastp), sickle (https://github.com/najoshi/sickle). Using trimmomatic (http://www.usadellab.org/cms/uploads/supplementary/Trimmomatic/TrimmomaticManual_V0.32.pdf) you should use the following options

- ILLUMINACLIP:${ADAPTERfasta}:2:30:3:1:true
- LEADING:20
- TRAILING:20
- SLIDINGWINDOW:3:15
- AVGQUAL:20
- MINLEN:35
- -summary ${outputfile}.summary

where ADAPTER.fasta is a file which contains a list of adapter sequences. Trimmomatic provides this for modern Illumina adapter sequences, however you should check that it includes those used by your sequencing facility especially if the data was generated some time ago. Trimmomatic performs the operations in the order listed, therefore if your reads have been trimmed previously and are potentially less than the MINLEN then you should apply the MINLEN twice, as the first and last operation to avoid errors. If fastq files are Phred+64 encoded you **must** use the following option to convert to Phred+33 encoding

- TOPHRED33

Alternatively, you can use seqtk (https://github.com/lh3/seqtk) to convert qscores.

NOTE: If fastq files contain reads that fail Illumina chastity these should also be removed.

NOTE: Should you have Illumina two color chemistry e.g. NovaSeq or NextSeq data you should also trim strings of G from the end of reads, these strings have normal qscores and so most trimming scripts will not trim them, they are however artefacts of the sequencing chemistry. Such sequences may be flagged by FastQC as over represented sequences or kmers. See https://sequencing.qcfail.com/articles/illumina-2-colour-chemistry-can-overcall-high-confidence-g-bases/

We highly recommend checking raw and filtered sequence reads with FastQC (http://www.bioinformatics.babraham.ac.uk/projects/fastqc/).

OFFICIAL

An example of trimmomatic command

```
java -jar /usr/local/trimmomatic/0.38/trimmomatic-0.38.jar PE -threads 8 -summary
${Fastq}.summary ${Fastq_R1}.fastq ${Fastq_R2}.fastq ${Fastq_R1}-trimmed.fastq
${Fastq_R1}-singleton.fastq ${Fastq_R2}-trimmed.fastq ${Fastq_R2}-singleton.fastq
MINLEN:${MINLEN} ILLUMINACLIP:${ADAPTER.fasta}:2:30:3:1:true LEADING:20 TRAILING:20
SLIDINGWINDOW:3:15 AVGQUAL:20 MINLEN:${MINLEN}
```

NOTE: the –summary ${Fastq}.summary option is not documented in the V0.32 pdf as it was added in V0.38. At the end of processing, the program prints to screen the results of the trimming. The –summary {file_name} option will also print these results to {file_name}. An example output is below. These data can be useful for evaluating the overall quality and levels of readthrough.

```
Input Read Pairs: 108316343
Both Surviving Reads: 97709067
Both Surviving Read Percent: 90.21
Forward Only Surviving Reads: 7903324
Forward Only Surviving Read Percent: 7.30
Reverse Only Surviving Reads: 1432840
Reverse Only Surviving Read Percent: 1.32
Dropped Reads: 1271112
Dropped Read Percent: 1.17
```

An example of FastQC command

```
/usr/local/bin/fastqc -q -t 12 *fastq.gz -o ${OUTPUTdirectory}
```

Reference Genome

ARS-UCD1.2_Btau5.0.1Y is the reference genome to be used in this project. This reference has the Btau5.0.1 Y chromosome assembly from Baylor College [48] added to ARS-UCD1.2 [27, 49]. It can be downloaded from the 1000 bull genomes website and https://sites.ualberta.ca/~stothard/1000_bull_genomes/ There are several files including the .fa.gz file (assembly) and checksums to ensure the download has not altered the files. This **exact** copy of the reference genome **must** be used to ensure your bam and GVCF files are compatible with the 1000 Bull Genomes Project pipeline. Non-conforming files will be excluded from the run.

Map fastq

Map trimmed reads (pairs and singles that pass above QC) to the reference using bwa mem (https://github.com/lh3/bwa) specifying read groups (see https://gatkforums.broadinstitute.org/gatk/discussion/6472/read-groups for definitions) with the following options

- -R @RG\\tID:${RGID}\\tPL:${RGPL}\\tLB:${RGLB}\\tSM:${RGSM}

where RGID is the sequencer lane (this is often within the fastq file name and is important for the base quality score recalibration steps later on), RGPL is the sequencing platform (ILLUMINA, SOLID or 454), RGLB is the library name and RGSM **must** be the international ID of the animal. Other read group tags can be populated but RGID, RGPL and RGSM are required. If your animal does not have an international ID, you should create one that conforms to Interbull standards, ie 3 character breed code + 3 character country code + sex code (M or F) + 12 character animal ID, eg HOLCANM000000352790. See http://www.interbull.org/ib/icarbreedcodes

To perform the subsequent steps you will need to use samtools sort to sort your bam and samtools index to index your sorted bam file (http://www.htslib.org/doc/samtools.html). Using the correct reference will ensure the bam files are sorted correctly, i.e. 1, 2, 3, …, 29, X, Y, MT, other contigs.

Where multiple bam files are generated for an individual you should use Picard MergeSamFiles (https://broadinstitute.github.io/picard/command-line-overview.html#MergeSamFiles) to merge them. Please note that samtools merge is not appropriate for individuals with multiple libraries as it doesn't handle the read groups properly, so Picard MergeSamFiles is our chosen tool for this task. The correct handling of libraries is important for downstream base quality score recalibration in GATK which is read group aware.

An example of bwa and samtools commands

```
/usr/local/bin/bwa mem -M -t 12 -R @RG\\tID:${RGID}\\tPL:ILLUMINA\\tSM:${RGSM} ARS-UCD1.2_Btau5.0.1Y.fa ${Fastq_R1}-trimmed.fastq.gz ${Fastq_R2}-trimmed.fastq.gz >
${OutputFile}-pe.sam
samtools sort -o ${OutputFile}-pe.sorted.bam -O BAM ${OutputFile}-pe.sam
samtools index ${OutputFile}-pe.sorted.bam
```

An example of Picard MergeSamFiles command

```
java -Xmx80G -jar  /usr/local/picard/2.1.0/picard.jar MergeSamFiles ${BAMlist} O=
${INTERNATIONALID}.sorted.bam VALIDATION_STRINGENCY=LENIENT ASSUME_SORTED=true
MERGE_SEQUENCE_DICTIONARIES=true
```

Mark Duplicates

Mark PCR and optical duplicates using Picard MarkDuplicates (https://broadinstitute.github.io/picard/command-line-overview.html#MarkDuplicates) with the following options

- VALIDATION_STRINGENCY=LENIENT
- OPTICAL_DUPLICATE_PIXEL_DISTANCE ${OPTICAL_DUPLICATE_PIXEL_DISTANCE}

where OPTICAL_DUPLICATE_PIXEL_DISTANCE is **100** for data generated on non-arrayed flowcells (ie from GAIIx, HiSeq1500/2000/2500), or **2500** for arrayed flowcell data (eg HiSeqX, HiSeq3000/4000, NovaSeq). Note these are all Illumina instruments, if you have data from other instruments you must work with the supplier to determine this value. See https://sequencing.qcfail.com/articles/illumina-patterned-flow-cells-generate-duplicated-sequences/

An example of Picard MarkDuplicates command

```
java -Xmx80G -jar /usr/local/picard/2.18.2/picard.jar MarkDuplicates
I=${INTERNATIONALID}.sorted.bam O=${INTERNATIONALID}_dedup.bam
M=${SAMPLE}_dedup.metrics
OPTICAL_DUPLICATE_PIXEL_DISTANCE=${OPTICAL_DUPLICATE_PIXEL_DISTANCE}
CREATE_INDEX=true VALIDATION_STRINGENCY=LENIENT
```

Base Quality Recalibration

Base quality recalibration should be performed according to GATK best practises guidelines (https://software.broadinstitute.org/gatk/guide/article?id=44). This task uses the GATK BaseRecalibrator (https://software.broadinstitute.org/gatk/documentation/tooldocs/3.8-0/org_broadinstitute_gatk_tools_walkers_bqsr_BaseRecalibrator.php) and PrintReads (https://software.broadinstitute.org/gatk/documentation/tooldocs/3.8-0/org_broadinstitute_gatk_tools_walkers_readutils_PrintReads.php) tools. Briefly, first GATK BaseRecalibrator is run to build a model of covariation based on the data and a set of known variants, to produce a recalibration table. Secondly, GATK PrintReads is run to adjust the base quality scores in the data based on the recalibration table, this produces a recalibrated bam. An optional third step runs GATK BaseRecalibrator on the recalibrated bam producing an "after recalibration"

table. These before and after recalibration tables can then be used to run GATK AnalyzeCovariates (https://software.broadinstitute.org/gatk/documentation/tooldocs/3.8-0/org_broadinstitute_gatk_tools_walkers_bqsr_AnalyzeCovariates.php) which generates plots that visualise the effects of the recalibration process (a recommended quality control step).

BaseRecalibrator requires the following options

- -knownSites:vcf ${KnownVariants}
- --bqsrBAQGapOpenPenalty 45

where bqsrBAQGapOpenPenalty has been tested and a value of 45 proves to work best for Bos whole genome sequencing (as opposed to default of 40 or recommendation of 30 for human). KnownVariants is a list of known variant sites in vcf format. We have generated two new known variants files.

1. ARS1.2PlusY_BQSR_v2.vcf.gz is a known variants file of SNP and INDEL generated from Bos Taurus and Bos Indicus Run7 at tranche 99.9 stringency. Please note that this known variants file has been extensively tested on *taurus* and *indicus* animals and is expected to work well. It is not recommended for *Bos* out species.
2. ARS1.2PlusY_BQSR_v3.vcf.gz combines ARS1.2PlusY_BQSR_v2.vcf.gz with variants called independently in various out species (see Appendix A for its derivation). This file has been extensively tested on *taurus*, *indicus*, bison (*Bison bison*), yak (*Bos grunniens*), gir (*Bos primigenius indicus*), gaur (*Bos gaurus*) and banteng (*Bos javanicus*) and is expected to work well. It has also been tested on water buffalo (Bubalus bubalis) for which it does not work well. It is always good to double check the QC metrics (see Appendix A for example of where BQSR has over corrected QV for Water Buffalo sequences) especially if you have animals from an out group not yet tested. Bob and Amanda would be happy to help with questions.

NOTE: We recommend using **ARS1.2PlusY_BQSR_v3.vcf.gz for all future submissions and --bqsrBAQGapOpenPenalty 45**, however if you have already used ARS1.2PlusY_BQSR_v2.vcf.gz for Taurus or Indicus animals there is no need to rerun. We also recommend that you check the before/after BQSR reports to ensure that samples are behaving as expected.

An example GATK BaseRecalibrator command

```
java -Xmx80G -jar $GATK -T BaseRecalibrator -nct 8 -R ARS-UCD1.2_Btau5.0.1Y.fa -I
${INTERNATIONALID}_dedup.bam -knownSites:vcf ${KnownSites} --bqsrBAQGapOpenPenalty
45 -o ${INTERNATIONALID}.recal.table
```

An example GATK PrintReads command

```
java -Xmx80G -jar $GATK -T PrintReads -nct 8 -R ARS-UCD1.2_Btau5.0.1Y.fa -I
${INTERNATIONALID}_dedup.bam -BQSR ${INTERNATIONALID}.recal.table -o
${INTERNATIONALID}_dedup_recal.bam
```

An example GATK AnalyzeCovariates command

```
java -Xmx80G -jar $GATK -T AnalyzeCovariates -R ARS-UCD1.2_Btau5.0.1Y.fa -before
${INTERNATIONALID}.recal.table -after after_recal.table -plots recal_plots.pdf
```

Create GVCF file

Appendix C explains a known issue with HaplotypeCaller and threading for the unmapped contigs.

Create a GVCF file using GATK HaplotypeCaller (https://software.broadinstitute.org/gatk/documentation/tooldocs/3.8-0/org_broadinstitute_gatk_tools_walkers_haplotypecaller_HaplotypeCaller.php). Follow the described "Single-sample GVCF calling on DNAseq (for `-ERC GVCF` cohort analysis workflow)" with the following options

- -ERC GVCF
- -variant_index_type LINEAR
- -variant_index_parameter 128000
- -o ${INTERNATIONALID}.g.vcf.gz

Where INTERNATIONALID is the international ID of the animal, NOTE: this INTERNATIONALID **must** match the international ID in the RGSM field in the read groups, which were added in the mapping steps above.

It is essential that GVCF files are gzipped and indexed (.tbi file). GATK will do both of these if you specify as above.

An example GATK HaplotypeCaller command

```
java -Xmx80G -jar $GATK -T HaplotypeCaller -nct 8 -R ARS-UCD1.2_Btau5.0.1Y.fa -I
${INTERNATIONALID}_dedup_recal.bam -o ${INTERNATIONALID}_dedup_recal.g.vcf.gz -ERC
GVCF -variant_index_type LINEAR -variant_index_parameter 128000
```

Calculate read coverage

It is important to know the coverage for a few reasons, one being to ensure compliance with the coverage requirements. Calculating coverage from the raw read numbers and length has been shown to be highly inaccurate of final coverage. Calculate the average read coverage using GATK DepthOfCoverage tool (https://software.broadinstitute.org/gatk/documentation/tooldocs/3.8-0/org_broadinstitute_gatk_tools_walkers_coverage_DepthOfCoverage.php). Include coverage statistic in the checklist (described below).

An example GATK DepthOfCoverage command

```
java -Xmx80G -jar $GATK -T DepthOfCoverage -R ARS-UCD1.2_Btau5.0.1Y.fa -I
${INTERNATIONALID}_dedup_recal.bam --omitDepthOutputAtEachBase --logging_level
ERROR --summaryCoverageThreshold 10 --summaryCoverageThreshold 20 --
summaryCoverageThreshold 30 --summaryCoverageThreshold 40 --
summaryCoverageThreshold 50 --summaryCoverageThreshold 80 --
summaryCoverageThreshold 90 --summaryCoverageThreshold 100 --
summaryCoverageThreshold 150 --minBaseQuality 15 --minMappingQuality 30 --start 1 -
-stop 1000 --nBins 999 -dt NONE -o ${INTERNATIONALID}_dedup_recal.coverage
```

****

Pipeline for joint processing of *taurus* & *indicus* sequences to vcf format

Processed by Agriculture Victoria, VIC 3083, Australia on behalf of Run8 1000 Bull Genomes Partners

Table 1 below gives details of the variant recalibration truth and training sets.

1) genotypeGVCFs

> java -Xmx450G -jar $GATK \
-R ARS-UCD1.2_Btau5.0.1Y.fa \
-T GenotypeGVCFs \

-V gvcfList.txt \
--disable_auto_index_creation_and_locking_when_reading_rods \
-o raw.vcf \
-log genotypeGVCF.log

2) Variant recalibration (SNP – note the same truth and training sets used as in Run7)

> java -Xmx450g -jar $GATK \
-R ARS-UCD1.2_Btau5.0.1Y.fa \
-T VariantRecalibrator \
-input raw.vcf \
-resource:HD,known=false,training=true,truth=true,prior=15.0 HD_truth.vcf \
-resource:GGPF250,known=false,training=true,truth=true,prior=15.0 GGPF250_truth.vcf \
-resource:Affy,known=false,training=true,truth=true,prior=12.0 Affy_truth.vcf \
-resource:1000bulls_truth,known=false,training=true,truth=true,prior=12.0 Run7_truth.vcf \
-resource:1000bulls_training,known=false,training=true,truth=false,prior=10.0
Run7_training.vcf \
-an QD -an MQ -an MQRankSum -an ReadPosRankSum -an FS -an SOR -an InbreedingCoeff -
maxNumTrainingData 10000000 \
-mode SNP \
-tranche 100.0 -tranche 99.9 -tranche 99.0 -tranche 90.0 \
-recalFile Run8_TAU.AS.recal \
-tranchesFile Run8_TAU.AS.tranches \
-rscriptFile Run8_TAU.plots.AS.R

3) Apply recalibration (SNP)

> java -Xmx230g -jar $GATK \
-R ARS-UCD1.2_Btau5.0.1Y.fa \
-T ApplyRecalibration \
-input raw.vcf \
-tranchesFile Run8_TAU.AS.tranches \
-recalFile Run8_TAU.AS.recal \
--ts_filter_level 90.0 \
-mode SNP \
-o Run8-TAU-applyRecal-SNP.vcf.gz

4) Variant recalibration (INDEL– note the same truth and training sets used as in Run7)

> java -Xmx450g -jar $GATK \
-R ARS-UCD1.2_Btau5.0.1Y.fa \
-T VariantRecalibrator \
-input Run8-TAU-applyRecal-SNP.vcf.gz \
-resource:1000bulls_truth,known=false,training=true,truth=true,prior=12.0
Run7_INDEL_truth.vcf \
-resource:1000bulls_training,known=false,training=true,truth=false,prior=10.0
Run7_INDEL_training.vcf \
-an DP -an QD -an MQRankSum -an ReadPosRankSum -an FS -an SOR -an InbreedingCoeff \
-mode INDEL \
-tranche 100.0 -tranche 99.9 -tranche 99.0 -tranche 90.0 \
--maxGaussians 4 \
-recalFile Run8_TAU_90-0_INDEL.AS.recal \
-tranchesFile Run8_TAU_90-0_INDEL.AS.tranches \
-rscriptFile Run8_TAU_90-0_INDEL.plots.AS.R

OFFICIAL

5) Apply recalibration (INDEL)

```
> java -Xmx230g -jar $GATK \
-R ARS-UCD1.2_Btau5.0.1Y.fa \
-T ApplyRecalibration \
-input Run8-TAU-applyRecal-SNP.vcf.gz \
-tranchesFile Run8_TAU_90-0_INDEL.AS.tranches
-recalFile Run8_TAU_90-0_INDEL.AS.recal \
--ts_filter_level 90.0 \
-mode INDEL \
-o Run8-TAU-applyRecal-SNP-INDEL.vcf.gz
```

6) For each animal, using custom scripts, generate QC metrics for variants falling in tranches 90.0, 99.0 and 99.9 (e.g. opposing homozygotes, heterozygosity, HD concordance, number unique variants).

7) Remove animals failing multiple QC metric thresholds (where to_remove.txt is a list of sample names for animals to be removed)

```
> bcftools view -Oz -S ^to_remove.txt Run8-TAU-applyRecal-SNP-INDEL.vcf.gz > Run8-
TAUapplyRecal-
SNP-INDEL.removed.vcf.gz
```

8) Remove any resulting monomorphic alleles

```
> java -Xmx10g -jar $GATK \
-R ARS-UCD1.2_Btau5.0.1Y.fa \
-T SelectVariants
--variant Run8-TAU-applyRecal-SNP-INDEL.removed.vcf.gz \
--excludeNonVariants \
-trimAlternates \
-o Run8-TAU-raw-toDistribute.vcf.gz
```

References

[55] Bellott DW, Hughes JF, Skaletsky H, Brown LG, Pyntikova T, Cho TJ, et al. Mammalian Y chromosomes retain widely expressed dosage-sensitive regulators. Nature. 2014 ;508:494-9.
[56] Rosen BD, Bickhart DM, Schnabel RD, Koren S, Elsik CG, Zimin A, et al. Modernizing the bovine reference genome assembly. In Proceedings of the 11th World Congress of Genetics Applied to Livestock Production : 11-16 February 2018 ; Auckland. 2018.

Table 2 – Run8 Taurus-Indicus truth and training sets (note that these truth and training sets are the same as those used for Run7)

Name (number variants)	Filters	Known	Training	Truth	Prior
Run7-TauInd-SNP-truth.vcf (9,914,679)	• Based on results from Run7 Taurus and Indicus animals processed using GATK's genotypeGVCFs tool and filtered using the traditional 1kbulls filtering thresholds (pre-Beagle correction).<br>• Additionally:<br>- MAF between 0.05 and 0.5<br>- At least 10 ALT homozygotes<br>- No flanking variants +/- 10bp<br>- No HWE p-value threshold<br>- Opposing homozygotes < 0.014	FALSE	TRUE	TRUE	12
Run7-TauInd-SNP-training.vcf (3,758,189)	• Based on results from Run7 Taurus and Indicus animals processed using GATK's genotypeGVCFs tool and filtered using the traditional 1kbulls filtering thresholds (pre-Beagle correction).<br>• As above but relaxed filters:<br>- MAF between 0.05 and 0.5<br>- At least 5 ALT homozygotes<br>- No flanking variants +/- 10bp<br>- No HWE p-value threshold<br>- Opposing homozygotes < 0.10<br>- Mutually exclusive to SNP above	FALSE	TRUE	FALS E	10
Affy_truth.vcf (323,782)	Comprised of filtered variants from the Affy pre-screening array prior to the building of the 600k Affy Bos-1 array. Selecting only variants with the frequency of "A" allele between 0.1-0.9. Converted to new reference using University of Missouri's ARS1.2 liftover files.	FALSE	TRUE	TRUE	12
GGPF250_truth.vcf (89,705)	Comprised of filtered variants from The University of Missouri based on 18,000 genotyped animals. Converted to new reference using University of Missouri's ARS1.2 liftover files.	FALSE	TRUE	TRUE	15
HD_truth.vcf (581,468)	Using our HD data. Iona's SNP that overlap with Run6. Converted to new reference using University of Missouri's ARS1.2 liftover files.	FALSE	TRUE	TRUE	15

* INDEL truth/training sets were the same as 1 and 2 above with 855,378 (Run7-TauInd-INDEL-truth.vcf) and 349,687 (Run7-TauInd-INDEL-training.vcf) INDELs respectively.

1000 Bulls BQSR Known Variants

Robert Schnabel & Amanda Chamberlain, October 2019

Base Quality Score Recalibration (BQSR) requires a large number of known variant sites in order to work effectively. For Run 8 we have created two known variants files, ARS1.2PlusY_BQSR_v2.vcf.gz and ARS1.2PlusY_BQSR_v3.vcf.gz. Both files used 1000 Bulls Taurus-Indicus Run7 variants (SNP and INDEL) from tranche99.9 stringency. Both files have been tested extensively by both Mizzou and Ag Victoria to verify that they produced recalibrated BAM files similar to, or superior to, what had previously been achieved using ARS1.2PlusY_BQSR.vcf.gz for *Bos Taurus* and *Bos Indicus* animals. Variants from unplaced contigs were excluded in both files.
