# Supplementary figures and images for: Empirical versus estimated accuracy of imputation: optimising filtering thresholds for sequence imputation
Source: Genet Sel Evol. 2024 Nov 15;56:72. doi: 10.1186/s12711-024-00942-2 (PMC11566673; doi:10.1186/s12711-024-00942-2)

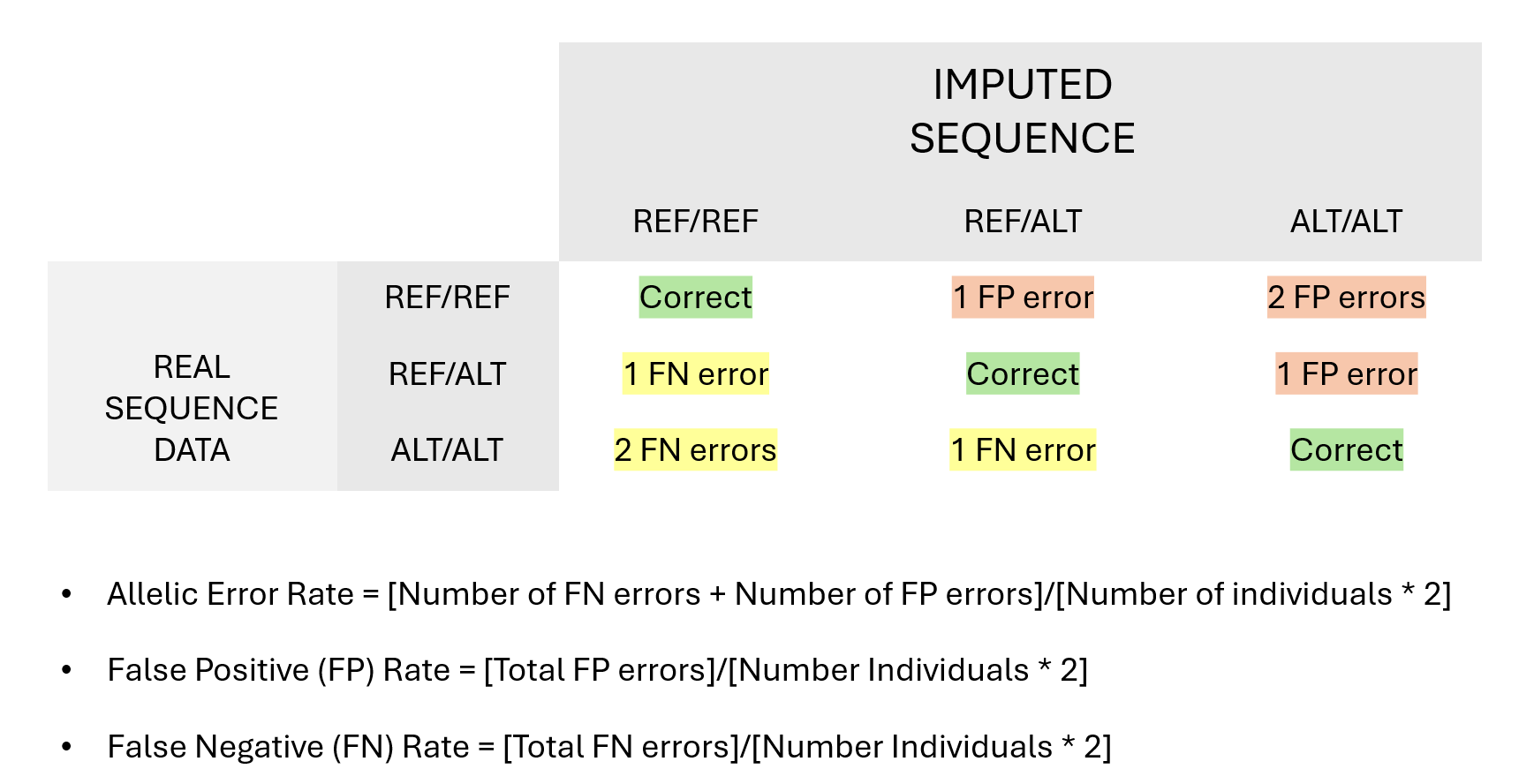

Supplement: Supplementary file 3 — Additional file 3: Graphical schematics of imputation cases used in the current analysis. REF represents the reference allele, ALT represents the alternative allele. [file 12711_2024_942_MOESM3_ESM.png]

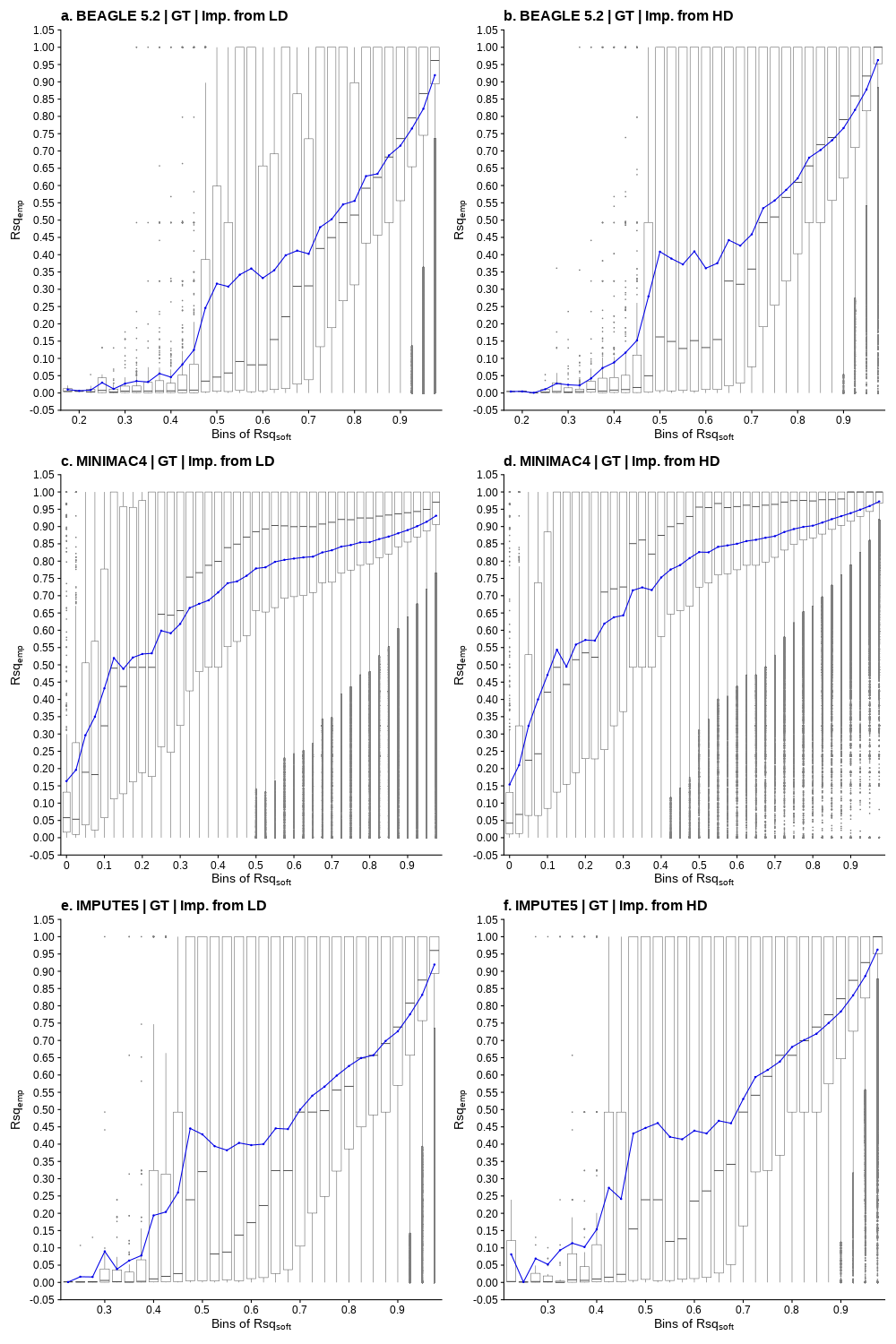

Supplement: Supplementary file 4 — Additional file 4: Boxplot visualizing relationship between Rsqsoft (Dosage R-squared - DR2) and Rsqemp (Empirical Correlation Squared) using imputed Genotype likelihoods (GT) on all chromosome tested using 3 programs Beagle 5.2, MINIMAC4 and IMPUTE5. The box contains the 25th to the 75th percentile of the data points. Whiskers extend to a maximum length of 1.5 times the interquartile range (IQR) beyond the box edges. Data points beyond the whiskers are represented by individual dots as outliers. (optional). [file 12711_2024_942_MOESM4_ESM.png]
